# Cutaneous UVB Irradiation Alters Gut Microbiota Independently of Skin Inflammation in SKH-1 Hairless Mice

**DOI:** 10.4014/jmb.2507.07020

**Published:** 2025-09-22

**Authors:** Eunjung Lee, Woo-Jin Sim, Tae-Gyu Lim

**Affiliations:** 1 Fermentation Convergence Research Group, Korea Food Research Institute, Wanju, Republic of Korea; 2 Department of Food Biotechnology, Korea University of Science and Technology, Daejeon, Republic of Korea; 3 Department of Food Science & Biotechnology, Sejong University, Seoul, Republic of Korea

**Keywords:** Ultraviolet B radiation, skin, gut microbiota, dysbiosis, *Lactobacillus johnsonii*, *Duncaniella freteri*

## Abstract

This study investigates the impact of chronic ultraviolet B (UVB) exposure on gut microbiota composition and skin inflammation. Mice were subjected to a 12-week regimen of low-dose UVB irradiation designed to mimic mild photoaging. Although no significant changes were observed at the phylum level, UVB exposure resulted in a significant decrease in the genus *Lactobacillus* (*P*=0.0302) and an increase in *Duncaniella* (*P*=0.046), accompanied by a reduction in α-diversity. At the species level, *Lactobacillus johnsonii*, a known probiotic, was significantly decreased, while *Duncaniella freteri* was increased in the UVB-exposed group. Despite these microbial alterations, no significant skin inflammation was detected, as mRNA levels of interleukin (IL)-1β and TNF-α and protein levels of IL-1β and IL-2 remained unchanged. These findings suggest that chronic low-dose UVB exposure can alter gut microbial homeostasis independently of local inflammatory responses, highlighting a potential role for the skin-gut axis in mediating systemic effects of environmental UVB exposure.

## Introduction

Ultraviolet B (UVB) radiation, with wavelengths ranging from 280 to 315 nm, primarily affects the epidermal layer of the skin, in contrast to UVA radiation, which penetrates more deeply [1]. Within the epidermis, the stratum corneum, basal keratinocytes, and melanocytes are the main targets of UVB exposure. UVB induces various cellular and molecular responses, including DNA damage, the generation of reactive oxygen species (ROS), and the production of pro-inflammatory cytokines [2, 3]. Although cells possess repair mechanisms to counteract UVB-induced damage, repeated or excessive exposure may overwhelm these systems. As a result, cells may undergo cycle arrest, activate aging- or apoptosis-related signaling pathways, and recruit immune cells [4]. This cascade can lead to the release of inflammatory mediators, immune cell infiltration, and tissue injury. Prolonged UVB exposure, particularly in the absence of sufficient repair, has been implicated in the development of photoaging and skin cancers [5, 6].

Advancements in metagenomic sequencing have expanded our understanding of the human microbiota, which consists of trillions of microorganisms residing across different body sites [7]. Among these, the gut microbiota plays a central role in regulating immune function, metabolism, and nutrient absorption [8]. Emerging evidence has highlighted a functional connection between the gut and skin, commonly referred to as the gut–skin axis [9, 10]. Alterations in gut microbial communities, known as dysbiosis, have been associated with a range of skin disorders, including acne vulgaris, atopic dermatitis, psoriasis, and rosacea [11-13]. The beneficial effects of certain probiotics, such as *Lactobacillus paracasei*, *Bifidobacterium longum*, and *Weissella viridescens*, further underscore the significance of the gut microbiota in supporting skin health [11, 14, 15].

Recent findings suggest that UVB exposure may influence gut microbial composition, even without direct light exposure to the gastrointestinal tract. Short-term exposure to narrow-band UVB (311 nm) has been shown to modulate the gut microbiota in both human and mouse models, alongside increases in circulating vitamin D levels [16-18]. However, the long-term impact of chronic UVB exposure on gut microbial composition and diversity remains unclear. In particular, the effects of repeated, low-level UVB exposure that does not visibly damage the skin have not been extensively studied. The biological effects of UVB are known to extend beyond the skin and may affect distant organs, including the gut, through systemic signaling. Understanding these skin-to-gut interactions is essential to assess the broader physiological consequences of UVB radiation. In this context, the present study aimed to investigate whether chronic exposure to low-dose UVB could alter gut microbial homeostasis. To eliminate confounding variables such as diet and fluctuations in vitamin D levels, we employed a mouse model under controlled environmental conditions.

Our experimental design focused on 12 weeks of mild UVB exposure, a duration and intensity insufficient to induce carcinogenesis but adequate to model subclinical photoaging. We hypothesized that this exposure regimen could disrupt the balance of the gut microbiota by reducing beneficial bacteria and promoting the growth of potentially harmful microbial populations. This study aims to provide new insights into the systemic consequences of chronic UVB exposure and the potential involvement of a skin–gut communication axis.

## Materials and Methods

### Animals and UVB Exposure

Six-week-old female SKH-1 hairless mice were purchased from Orient Bio (Republic of Korea) and divided into 2-3 mice per cage under standard laboratory conditions. The animal facility was maintained at a constant temperature of 23°C with a 12-h light-dark cycle (lights on from 7:00 AM to 7:00 PM; lights off from 7:00 PM to 7:00 AM). All animals had *ad libitum* access to standard laboratory chow (Teklab 2018S diet, Harlan Laboratory, USA) and water. After a 1-week acclimation period, the mice were randomly assigned to either a UVB exposure group or a control group (*n* = 10 per group). UVB irradiation was administered three times per week for 12 weeks, with gradually increasing doses: 1 minimal erythema dose (MED) during the first week, 2 MEDs in the second week, 3 MEDs in the third week, and 4 MEDs from the fourth week onward. A single MED was defined as 0.05 J/cm^2^, following previous study [19]. UVB was delivered using UVB lamps (Sankyo, Japan) emitting wavelengths in the 280-360 nm range. The lamp was positioned 30 cm above the dorsal skin, and exposure times were adjusted to deliver the desired dose. UV intensity was measured with a UV radiometer (Viber, France) to ensure accurate dosing. Control mice were placed in the UVB chamber without irradiation (light off) for the same duration as the UVB-treated group each week. Between cage-level exposures, the chamber was cleaned with 70% ethanol and irradiated without mice to minimize residual contaminants and maintain a clean environment. Throughout the study, body weight and food intake were monitored regularly. In the final week of the experiment, transepidermal water loss (TEWL) and stratum corneum hydration (SCH) were measured using a gpskin (GPOWER, Republic of Korea). At the end of the experiments, mice were anesthetized and taken serum and tissues. All procedures involving animals were reviewed and approved by the Institutional Animal Care and Use Committee of Korea Food Research Institute (Approval number: KFRI-M-21040).

### Fecal Sample Collection and Analysis

Because mice in a cage may share gut microbiota due to the habits of mice (*e.g.*, coprophagy), feces were pooled as a signle sample from 2-3 mice of same cage in sterile tubes (*n* = 4 per group), immediately placed on ice and stored at -80°C until further analysis. Total metagenomic DNA extraction and sequencing were conducted by Macrogen Inc. (Republic of Korea). DNA was extracted using a DNeasyPowerSoil Pro Kit (Qiagen, Germany) and quantified using Quant-IT PicoGreen (Invitrogen, USA) according to the manufacturer's instructions. For library construction and sequencing, the PacBio amplicon Template Preparation and Sequencing protocols were followed, targeting the 27F-F (5’-AGRGTTYGATYMTGGCTCAG-3’) and 1492-R (5’-RGYTACCTTGTTACGACTT-3’) regions. The initial gDNA (2 ng) underwent PCR amplification with TaKaRa LA Taq (Takara, Japan) using specific F/R PCR primers and reagents. PCR conditions included an initial heat activation at 94°C for 5 min, followed by 35 cycles of denaturation at 94°C for 30 sec, annealing at 53°C for 30 sec, and extension at 72°C for 90 sec, followed by a final extension at 72°C for 5 min. Amplicons were purified with AMPure beads, quantified with Quant-IT PicoGreen (Invitrogen), and qualified using TapeStation D5000 Screen Tape (Agilent Technologies, Germany). Library preparation for PacBio Sequel sequencing utilized 500 ng of pooled amplicon DNA. The PacBio SMRTbell Express Template Prep Kit 2.0 was employed, followed by annealing and binding using the Sequel Binding and Internal Ctrl Kit 3.0. Sequencing was conducted with Sequel Sequencing Kit 3.0 and SMRT cells 1M v3 Tray and performed on the PacBio Sequel platform (Pacific Biosciences) using 10-h movies for each SMRT cell. Further steps were executed according to the PacBio Sample Net-Shared Protocol, accessible at http://pacificbiosciences.com.

### Data Processing and Multivariate Analysis

Multivariate statistical analysis was performed using the SIMCA-P 14 Software (version 14.0, Sweden) on the fecal metagenomic dataset. All data were subjected to Pareto scaling to facilitate discrimination between the control and UVB-exposed groups. Principal component analysis (PCA), an unsupervised pattern recognition method, was used to reveal intrinsic variation within the dataset. In addition, orthogonal partial least squares discriminant analysis (OPLS-DA), a supervised classification method, was applied to distinguish between two experimental groups. Model quality was assessed by examining R^2^X and Q^2^ values obtained through permutation from the PLS-DA plot. R^2^X represents the proportion of variance in the data explained by the models, indicating a good fit, and Q^2^ denotes the proportion of variance in the data predictable by the model, signifying its predictability. From the OPLS-DA, an S-plot and variable influence on projection (VIP) scores were obtained to identify key features contributing to group separation. Variables with VIP scores greater than 1.0 were considered significant contributors to discrimination between the groups.

### Quantitative Real-Time Polymerase Chain Reaction (qPCR) Analysis

The RNA extraction from skin tissues was carried out using TRIzol reagent (Thermo Fisher Scientific, USA), yielding 1 mg of total RNA. Subsequently, cDNA was synthesized from this RNA using the amfiRivert cDNA Synthesis Platinum Master Mix (GenDEPOT, USA). Quantitative PCR (qPCR) was performed in accordance with the StepOne Plus manufacturer’s instructions (Applied Biosystems), employing Fast SYBR Green Master Mix (Life Technologies, USA). The quantification of IL-1β and TNF-α mRNA levels was accomplished using specific primers: IL-1β-F (5’-TGGACCTTCCAGGATGAGGACA-3’), IL-1β-R (5’-GTTCATCTCGGAGCCTGTAGTG-3’), TNF-α-F (5’-GGTGCCTATGTCTCAGCCTCTT-3’), and TNF-α-R (5’-GCCATAGAACTGATGAGAGGGAG-3’). GAPDH (F: 5’-CATCACTGCCACCCAGAAGACTG-3’, R: 5’-ATGCCAGTGAGCTTCCCGTTCAG-3’) was employed as an endogenous control to normalize the mRNA expression in mouse samples. All procedures strictly followed the respective manufacturer’s guidelines.

### Western Blotting

Ten skin tissues from each group were prepared and subsequently underwent western blot analysis, following the established protocol [20]. Following the blotting process, a polyvinylidene difluoride membrane (Thermo Fisher Scientific) was incubated overnight at 4°C with primary antibodies against IL-1β (Thermo Fisher Scientific), IL-2 (Bioss Antibodies, USA) and Vinculin (Santa Cruz Biotechnology). Protein bands were visualized through Clarity Western ECL Substrate from Bio-Rad (USA), and the LuminoGraph 3 Lite (Atto, Japan) at the Biopolymer Research Center for Advanced Materials was employed for visualization after hybridization with a horseradish peroxidase-conjugated secondary antibody. The quantification of band intensity was performed using Image J software (version 1.54d, National Institutes of Health, USA).

### Statistical Analysis

All graphs express mean ± SEM. values and were analyzed using Student’s *t*-test. A probability value of *p* <0.05 was used as the criterion for statistical significance. All analyses were performed using Prism software (Graphpad Software, USA).

## Results and Discussion

### The Impact of Chronic Exposure of UVB to Skin on Gut Microbial Composition and Diversity

**Fig. 1:**
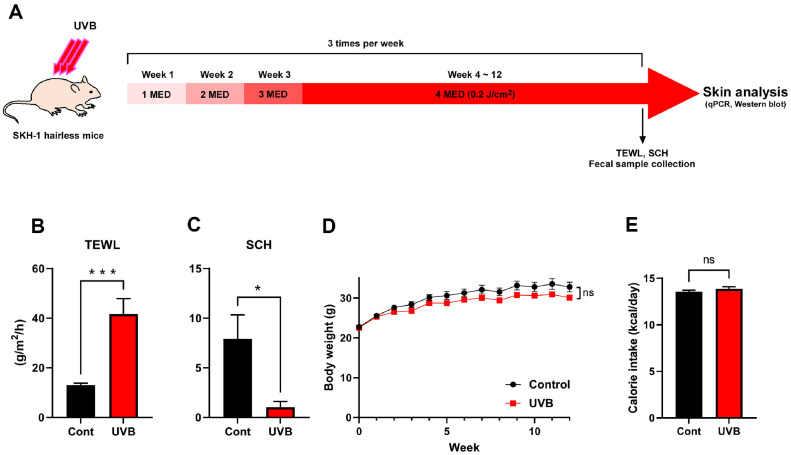
Experimental design and physiological effects of chronic UVB exposure in SKH-1 hairless mice. (**A**) Schematic representation of the UVB exposure protocol. (**B–C**) Skin barrier function was assessed by measuring TEWL (**B**) and SCH (**C**). (**D**) Body weight was measured weekly during the 12-week period. (**E**) Daily caloric intake. Data are presented as mean ± SEM (*n* = 10 per group). *P < 0.05, ***P < 0.001, ns: not significant. TEWL, transepidermal water loss; SCH, stratum corneum hydration.

In this study, we employed a broader UVB spectrum (280–360 nm) to assess its potential impact on gut microbiota composition, in contrast to previous studies [17, 18] that primarily focused on narrow-band UVB (NB-UVB) exposure. The UVB exposure regimen was designed to induce mild photoaging effects, such as wrinkle formation and flushing, with doses ranging from 0.05 to 0.2 J/cm^2^ administered three times per week for 12 weeks (Fig. 1A). After 12 weeks, TEWL increased and SCH decreased (Fig. 1B and 1C), confirming that the UVB treatment partially induced photoaging-like skin changes, consistent with previous reports. No significant differences were observed in body weight (Fig. 1D) or caloric intake (Fig. 1E), indicating that the UVB exposure did not cause systemic adverse effects.

**Fig. 2:**
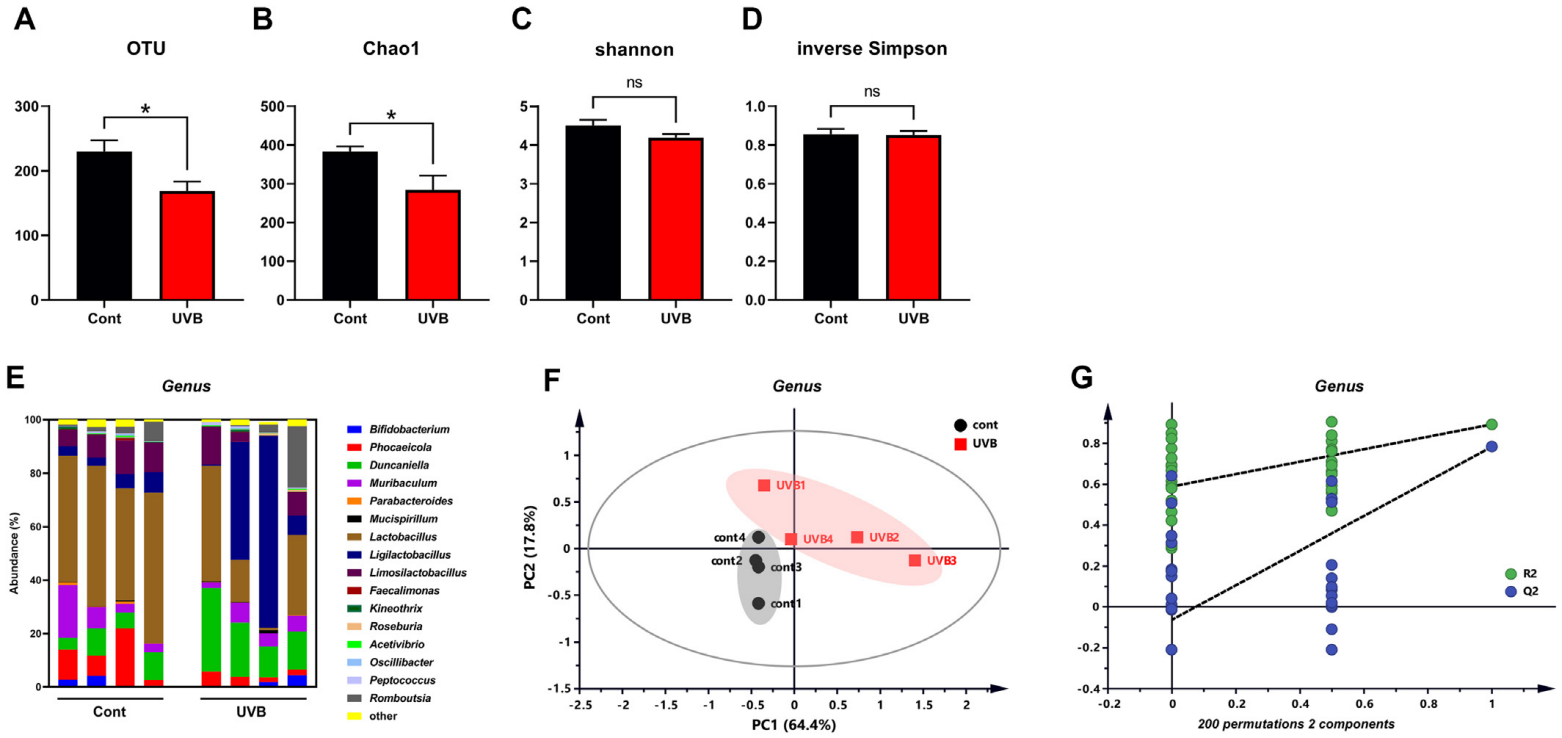
Impact of chronic UVB exposure into skin on gut microbial composition and diversity. (**A-D**) Alpha diversity indices of fecal microbiota. Comparison of alpha-diversity indices of gut microbial communities between the two groups. Data are presented as mean ± SEM. (**E**) Abundance of fecal microbiota at the genus levels, (**F**) PCA plot based on the taxonomic abundance data of genus levels from the fecal samples of control (black circle) and UVB-exposed mice (red square) (**G**) Permutation test with 200 permutations of the PLS-DA model, R2 = (0.0, 0.589), and Q2 = (0.0, -0.0642). Significant differences between groups are indicated by asterisks (*P < 0.05). PCA, Principal Component Analysis; PLS-DA, partial least square discriminant analysis; OTUs, observed taxonomic units.

As shown in Fig. 2A and 2B, both the observed taxonomic units (OTUs) and Chao1 richness index significantly decreased following UVB exposure. In contrast, the Shannon and Simpson indices remained unchanged (Fig. 2C and 2D), suggesting a reduction in microbial richness but not in community evenness. This finding reflects a reduction in alpha diversity of the gut microbiota, highlighting the impact of chronic UVB exposure on microbial ecology. At the genus level, the relative abundance of *Lactobacillus* significantly decreased from 49.50 ± 0.03% in the control group to 22.50 ± 0.09% in the UVB-exposed group (*P* = 0.0302, Fig. 2E), while *Duncaniella* significantly increased from 7.75 ± 0.01% to 19.39 ± 0.04% (*P* = 0.046). PCA based on genus-level profiles revealed distinct clustering between the two groups (Fig. 2F), indicating clear differences in microbial composition. Furthermore, permutation testing (*n* = 200) in the corresponding PLS-DA model yielded significantly lower R^2^ and Q^2^ values in the permuted datasets than in the original model (Fig. 2G), validating the model’s ability to differentiate the groups.

Interestingly, in contrast to previous NB-UVB studies that consistently reported an increase in Firmicutes and a decrease in *Bacteroidetes* [16, 17], our study found no significant changes at the phylum level after 12 weeks of UVB exposure (data not shown). This discrepancy may result from differences in UVB spectral range and sequencing platforms. Notably, while previous studies utilized Illumina-based short-read sequencing, we employed the PacBio long-read platform, which allows for higher resolution taxonomic classification. These initial findings indicate that chronic low-dose UVB exposure can modulate gut microbial diversity and composition at the genus level, even in the absence of systemic physiological disturbances.

### Chronic UVB Exposure Alters Species-Level Composition of the Gut Microbiota

**Fig. 3:**
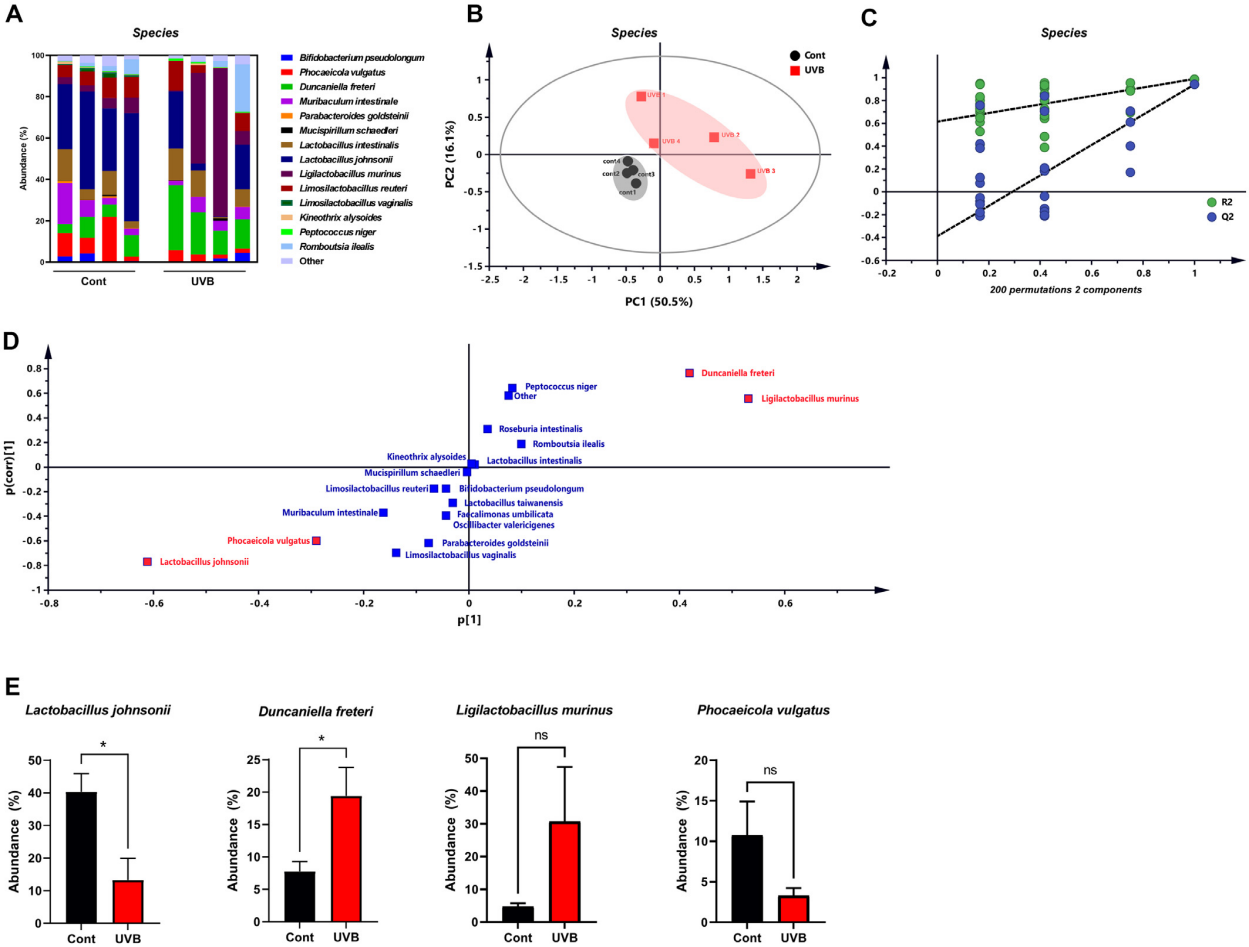
Chronic UVB exposure alters species-level composition of the gut microbiota. (**A**) Relative abundance of fecal microbiota at the species level. (**B**) PCA plot based on species abundance data, illustrating distinct clustering between control (black circle) and UVB-exposed mice (red square). (**C**) Permutation test with 200 iterations in a PLS-DA model based on species-level abundance, indicating R^2^ = (0.0, 0.615) and Q2 = (0.0, -0.389) in the permuted analysis, both significantly lower than those of the original model. (**D**) S-plot derived from the OPLS-DA analysis. Red-marked data points indicate the four bacterial species with the highest variable importance in projection (VIP) values, contributing most significantly to group separation. (**E**) Relative abundances of the top four species with high VIP scores. Values are expressed as mean ± SEM. *P < 0.05. OPLS-DA, orthogonal partial least squares discriminant analysis; ns, not significant.

Following genus-level analysis, species-level profiling further revealed distinct compositional differences between control and UVB-exposed groups. Fecal metagenomic analysis identified significant divergence in species richness (Fig. 3A). Principal component analysis (PCA) based on species-level abundance confirmed clear separation between the two groups (Fig. 3B), which was validated by permutation testing (Fig. 3C). Orthogonal partial least squares discriminant analysis (OPLS-DA) was subsequently performed, and the corresponding S-plot (Fig. 3D) and variable importance in projection (VIP) scores identified nine bacterial species with VIP > 1.0, indicating their strong contribution to group differentiation. Among these, *Lactobacillus johnsonii* was significantly decreased, while *Duncaniella freteri* was significantly increased in the UVB-exposed group (Fig. 3E). The remaining seven species, including *Ligilactobacillus murinus* and *Phocaeicola vulgatus*, did not show statistically significant changes in relative abundance.

*L. johnsonii* is a well-characterized member of the *Lactobacillus* genus and is widely recognized for its probiotic properties. Previous studies have shown that specific strains of *L. johnsonii* enhance intestinal barrier function via induction of cytoprotective heat shock proteins (HSPs) and inhibition of enterotoxigenic *Escherichia coli* (ETEC) adhesion [21]. Additionally, co-administration of *L. johnsonii* La1 with antioxidants has been reported to prevent bacterial translocation in cirrhotic mice, thereby reducing endotoxemia [22]. In elderly populations, *L. johnsonii* supplementation has been shown to reduce infection frequency, improve nutritional status, and enhance innate immune responses [23]. In contrast, *D. freteri*, a recently identified species, remains poorly understood in terms of its role in host physiology [24]. Its abundance has been shown to decrease in mice treated with *Saxifraga stolonifera* Curt, a herb with anti-inflammatory properties [25], while it increased in a murine mammary tumor xenograft model [26]. Interestingly, the species-level analysis showed that *L. johnsonii* decreased and *D. freteri* increased. Along with the observed decrease in alpha diversity (Fig. 2A), these results suggest that chronic UVB exposure may contribute to gut microbial dysbiosis at multiple taxonomic levels.

### Low-Dose UVB Exposure Does Not Induce Skin Inflammation but May Affect Gut Microbiota Independently

**Fig. 4:**
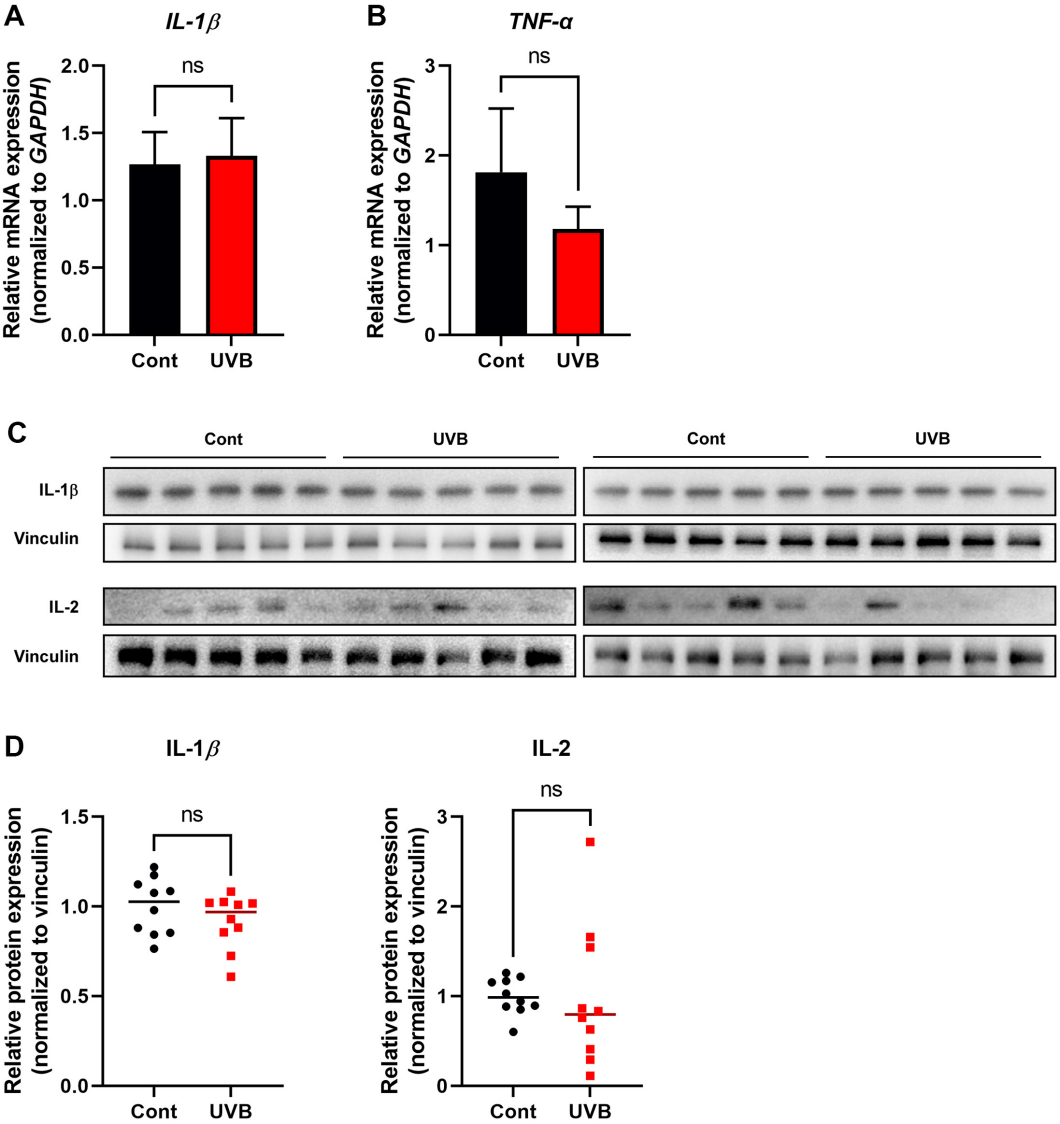
Chronic UVB exposure does not significantly induce inflammatory responses in skin tissues. (**A, B**) mRNA expression levels of pro-inflammatory markers *IL-1β* (**A**) and *TNF-α* (**B**) in skin tissues, measured by qPCR. (**C**) Protein levels of IL-1β and IL-2 assessed by western blot analysis (*n* = 10). (**D**) Quantified band intensities were represented by individual dots (control in black circle, UVB in red square) with a line indicating the mean. ns, not significant. Values are expressed as mean ± SEM. ns, not significant.

Twelve weeks of low-dose UVB exposure resulted in significant alterations in gut microbiota composition, prompting investigation into whether these changes could be associated with skin inflammation. To assess the potential inflammatory response in the skin, mRNA expression levels of representative inflammatory markers IL-1β and TNF-α were measured (Fig. 4A and 4B). No significant differences were observed between the UVB-exposed and control groups. Additionally, western blot analysis showed no changes in protein expression levels of IL-1β and IL-2, which are commonly associated with UVB-induced skin inflammation and tumorigenesis (Fig. 4C and 4D). These results suggest that the UVB exposure protocol used in this study—up to 4 minimal erythema doses (MED, 0.2 J/cm^2^) administered three times per week for 12 weeks—represents a mild regimen that does not elicit significant cutaneous inflammation. Notably, this implies that dysbiosis of the gut microbiota observed in this study may occur independently of overt inflammatory responses in the skin, potentially preceding or even contributing to systemic inflammatory processes.

In contrast to our findings, a recent study reported increased expression of inflammatory markers and macrophage infiltration in the kidneys following chronic UVB exposure [27]. However, key methodological differences exist, including mouse strain (shaved C57BL/6N vs. hairless SKH-1), MED definition (0.1 vs. 0.05 J/cm^2^), and UVB exposure duration and intensity (14 weeks, up to 0.3 J/cm^2^). When scrutinizing previous research utilizing similar strains, it becomes evident that the level of UVB exposure in our study is below the threshold required to elicit a substantial inflammatory response [28, 29]. Taken together, our findings indicate that the relatively low-dose UVB regimen used in this study does not induce measurable skin inflammation, yet is associated with distinct alterations in gut microbial composition. While these observations are consistent with the hypothesis that chronic UVB exposure may influence systemic health via a skin-gut axis, we acknowledge that our current data are correlative and do not establish a direct mechanistic link. Thus, further investigation is required to determine whether skin photoaging-related pathways or UVB-induced signaling in the skin contribute causally to the observed microbial shifts.

## Conclusion

In summary, this study demonstrates that chronic, low-dose UVB exposure to the skin can alter gut microbiota composition and reduce α-diversity, even in the absence of overt skin inflammation. These findings suggest a potential role for the skin-gut axis in mediating UVB-induced effects on host health, independent of classical inflammatory responses. Further studies are warranted to elucidate the underlying mechanisms and long-term physiological relevance of this interaction.
